# The Sävsjö-school-project: a cluster-randomized trial aimed at improving the literacy of beginners—achievements, mental health, school satisfaction and reading capacity at the end of grade three using an alternative school curriculum

**DOI:** 10.1186/s13034-019-0285-0

**Published:** 2019-06-24

**Authors:** Göran Ahlqvist, Jan-Olov Larsson, Tatjana von Rosen, Mara Westling Allodi, Per-Anders Rydelius

**Affiliations:** 1 Department of Special Education, Stockholm University, 106 91 Stockholm, Sweden; 2 Department of Women’s and Children’s Health, Karolinska Institutet, 171 77 Stockholm, Sweden; 3 Department of Statistics, Stockholm University, 106 91 Stockholm, Sweden

**Keywords:** Childhood intervention, School mental health, primary prevention program, School start, Long-term effects

## Abstract

**Background:**

A curriculum was planned using modern concepts based on the “old” principles to test if such an educational intervention provided pupils with good mental health and a solid basis for good reading and writing skills, as well as generated a positive attitude to learn. These “old” principles were based on previous knowledge derived from school psychiatry (which in Sweden was a branch of child and adolescent psychiatry 1915–1970), educational psychology and the educational approach from the differentiating Swedish School system of 1946–1970 (itself based on the principles of curative education “Heilpädagogie”, which was later renamed mental health care).

**Methods:**

All six available schools in the small Swedish city of Sävsjö participated in the study. In these six schools there were eight preschool classes that included every 6-year old child living in the city. In total there were 184 families with 186 children (including 2 pairs of twins) who belonged to these preschool classes and were invited to take part in the study. One family moved just before school-start and 8 decided not to participate, thus 177 children (84 boys and 93 girls, aged 5.6–6.6 years) entered the study. The preschool classes were randomized into an experimental group with four preschool classes and a comparison group with four preschool classes. The experimental group followed a teaching program from the start of the preschool year until the end of grade 3 that was tailored to each student’s individual capacity based on the concepts of school maturation and curative education used in the Swedish schools during the period 1946–1970. The comparison group followed today’s average Swedish school curriculum. The project was planned as an intervention study covering the preschool year and the first 3 years of elementary school, which was to form a basis for a follow-up when the pupils had left senior high, the 12th year in Swedish public school. The outcome and the achievements were measured at end of grade 3 using standardized tests on reading, writing and mathematical skills. Behavior was assessed at school start and at end of grade 3 using the Child Behavior Check List (CBCL-scales) in addition to a questionnaire on Attention Deficit Hyperactivity Disorder (AD/HD) with criteria from DSM-IV. The children made a self-evaluation of their attitude towards learning.

**Results:**

At the end of school year 3, the children in the experimental group had an improved reading capacity (p = 0.002, effect size(es) = 4.35) and reading comprehension (p = 0.03, es = 0.04). They evaluated their own reading (p = 0.02, es = 0.23), writing (p = 0.007, es = 0.35) and mathematical skills (p = 0.003, es = 0.48) as going “very well” when compared to comparison group. Differences regarding intelligence quotas between the groups at the start of school had disappeared by the end of grade 3. No differences referring to CBCL were found at end of grade 3. One child in the comparison group fulfilled criteria for AD/HD, according to parents and teachers.

**Conclusions:**

The alternative curriculum covering the preschool year through the first 3 years of elementary school based on the old principles from curative education (“Heilpädagogie”), educational psychology and school psychiatry gave the children in the experimental group a better reading capacity and reading comprehension.

*Trial registration* The study started in 1998. The data were collected longitudinally and prospectively but have not been analyzed until now, with the children having left senior high. A retrospective registration in the ISRCTN is pending.

## Introduction

The Sävsjö project’s theoretical and empirical background and design have been described previously in Swedish [1, 2] and are summarized as follows: A resumed interest in Sweden for Dyslexia made 1997 “the year of Dyslexia”, a time when special promotions were made to give children and adolescents suffering from dyslexia better support in school. The Swedish Disability Ombudsman designated the city of Sävsjö as one of Sweden’s pilot communities for the establishment of better help for children and youth with hidden disabilities, i.e. learning disabilities, slow learning, dyslexia, and dyscalculia [1].

### Aim of the study

Due to a joint interest from the Sävsjö (English spelling = Savsjo) city council, the city’s school authorities and researchers in special education and child and adolescent psychiatry, the project planned to determine if during preschool through the first 3 years of elementary school an alternative curriculum (based on the old principles of curative education (“Heilpädagogie”), educational psychology and school psychiatry previously used in the Swedish Public School) could:

- Improve the students’ cognition, linguistic awareness and social competence.
- Lay foundations for good reading and writing skills.
- Create a positive attitude towards learning and a good collaboration with other students and teachers, in order for each student to be integrated/included in the social community of the class and share the class´ common level of knowledge and be part of the “knowledge community” in the class to avoid exclusion.
- Result in better school achievements for all school children through the end of senior high.

### Theoretical and empirical background

Based on previous knowledge of school psychiatry (which in Sweden was a branch of child and adolescent psychiatry 1915–1970), and the educational approach of the differentiating Swedish School system during the period 1946–1970 (based on the principles of curative education and educational psychology), an alternative curriculum was planned using modern concepts of the principles recommended by the Swedish Education Commission in 1946 [3]. These principles included school maturity testing (the Swedish concept for school-readiness)—in order to emphasize the concept of school-readiness—the value of small groups and individualized teaching in class, and the need for children with slow learning to be taught by teachers trained in special education.

School psychiatry was developed as a branch of the expanding Swedish child and adolescent psychiatry. In 1915, the very first outpatient unit for child and adolescent psychiatry in Sweden opened in the Stockholm public school system to support children with learning difficulties. A special interest in the area of school psychiatry existed between 1915 and 1970. The focus was to establish an integrated approach to pupils’ mental health and their school achievements. These pioneering Swedish school psychiatrists observed that children with learning problems of different kinds usually displayed mental problems and behavioral symptoms in class if the teaching was not adapted to their learning capacity and/or their specific learning problems. Children with slow learning abilities, “slow learners”, (IQ = 70–90), which until 1973 was considered mental retardation) showed restlessness, impulsivity and a difficulty concentrating in such situations [4–6].

During this period of time, in both Swedish Child and Adolescent Psychiatry and Swedish Education, the concept of curative education (“Heilpädagogie”) [7, 8] was very important. Until the 1950s, curative education was one of the main clinical paradigms in Swedish Child and Adolescent Psychiatry to assess and treat children with mental retardation, behavioral disturbances and school problems. In the early 1950s, the terminology was changed from “curative education” to “mental health care” and “school mental health care”.

The principles of curative education were used by child and adolescent psychiatrists to assess the child’s overall physical and mental state, including development and his/her strengths and difficulties, as accurately as possible in order to support his/her strengths and minimize his/her difficulties as efficiently as possible.

In the school system, the teachers used the principles in a similar way to assess the pupils’ strengths and difficulties with regard to behavior and learning in order to individualize teaching and treatment in the classroom with special consideration to his/her strengths and difficulties. IQ-tests and tests to assess reading and writing skills, as well as mathematical skills, were developed for teachers to use, and educational tools were developed to facilitate teaching.

Textbooks based on the principles of curative education and school mental health were published [9–13] to train teachers, psychologists and child and adolescent psychiatrists. Torsten Ramer, one of the pioneering Swedish school psychiatrists presented the use of these principles in a more comprehensive way in order to prevent mental problems among school children [14].

Pioneering research in school psychiatry was performed. At the Mellansjö School-home, which was managed from 1928 to 1956 by Alice Hellström (a teacher, MD and pioneer of child- and adolescent psychiatry), an approach based on curative education was used to support psychopathic children and children who had failed at regular school. Some of the children who came to the treatment-home were extremely hyperactive. Karin Koch, one of the teachers, used a special program for this group. In 1941 she wrote about the “Practical Class” [6]: “The practical class comprises nine children characterized by extreme motor restlessness. They have an IQ of 89–134 and are aged between 11 and 13 years. The Practical Class is an experiment, and it was started because these children used to disturb the work of the quieter children. There was also a desire to see if another method of work would stimulate restless children who were tired of school to do some kind of work—any kind. Then perhaps they could continue once they had grown accustomed to the “work swing to do more orderly schoolwork”. The schooldays were based on shorter lessons, and regular teaching was individualized and given parallel to other activities, so-called “practical work”.

She described the outcome as follows: “The children have become calmer, less talkative, more ambitious and more friendly with one another”. In 1946, Torsten Ramer [5] presented his thesis “The Prognosis of the Mentally Retarded” focusing on the school outcome of “slow learners”, i.e. children with IQ 70–90. Also, in 1950, Bertil Hallgren [15] published his thesis “Specific Dyslexia (“Congenital Word-Blindness”): A Clinical and Genetic Study” showing that approx. 5% of children with dyslexia had “Specific Dyslexia” with a hereditary origin.

Swedish researchers and clinicians in child and adolescent psychiatry, psychology and education paid considerable attention to the normal and great variation in growth, especially with respect to “learning age” in relation to the “chronological age” in average children, and how this can change over time and influence teaching. The concepts of growth, maturation and development are used here, as growth and maturation reflect mainly “nature” in the nature-nurture concepts while development includes “nurture”.

Based on the findings by Honzik, Macfarlane et al. [16], which show that IQ may increase during growth by an average of 15 IQ points, Malmquist [17] discussed the challenges for teachers following the findings that “learning age” (mental age) in average children at the chronological age of 12 years may vary from 9 to 18 years.

Husén [18] referred to Willard C. Olson’s studies on “Child Development” [19] in showing how reading capacity and IQ can change over time. He referred specifically to Olson’s finding of late maturing children who “surprise us with their later achievements” commenting that “their fate lies in the hands of caring parents and teacher who have kept the way open”. In 1965, Bengt-Olov Ljung, one of Husén’s Ph.D. students, presented his thesis on “The adolescent spurt in mental growth” [20]. These previous findings were recently supported by Ramsden et al. [21] in their findings that “Verbal and non-verbal intelligence changes in the teenage brain”.

Growth and maturation, “nature”, reflect the child’s capacity to change over time based on his/her genes and constitution while development, “nurture”, reflects effects of care and treatment. Husén and Tuijnman [22] found in a longitudinal study of a male Swedish cohort that “formal schooling is enhancing the intellectual capital of a nation”. This can illustrate the complex interaction between nature and nurture when discussing the effects of education.

School psychiatric teams were set up in the regular schools to assess children and to cooperate with and guide teachers in their activities when dealing with children in need of special support. In the larger cities these teams included a school psychiatrist, a school psychologist, a social worker and a special pedagogue. They worked with the following pupils:

- Children with specific learning difficulties i.e. dyslexia, dyscalculia.
- Children with MR, IQ < 70.
- Slow learners, i.e. children with IQ 70–90.
- Children with slow maturation.
- Talented children.
- “Original” children (including some children with high functioning autism spectrum disorders).
- Children with behavior disturbances of other reasons.

The Swedish school system, as based on differentiation from IQ-levels, was used 1946–1970. It was thereafter heavily criticized, and from 1970 it was replaced by a public school based on the concept of inclusion. Four of the more experienced professors in psychology and education wrote a pamphlet in 1959 [18] discussing the negative effects of the differentiation of pupils in preparation for change. However, in Husén’s chapter [18] there is an interesting discussion on teaching using “differentiation within the class”. This gives an opportunity to have different teaching groups within the same class in order to support weak pupils and avoid exclusion. This concept of “differentiation within the class” was used by us in this project.

## Methods

### The design of the study

The study was designed as a randomized controlled intervention study (a cluster-randomized trial) with an experimental group and a comparison group. The intervention covered 4 years from the start of the preschool class until the end of grade 3 in elementary school. From grade 4 it was planned to form the basis for a follow-up at the end of senior high (the 12th grade in the Swedish school system).

Sävsjö (Savsjo) is a small city with a city center, the main town, and surrounding rural areas (three smaller suburbs). When the project started there were 10 986 inhabitants living in the city. The city population had an age distribution similar to the local county area (Jönköping County) and to Sweden as a whole. Among the families and children entering the project, 97% of the children, 92% of the mothers and 94% of the fathers were born in Sweden.

#### School setting and randomization

When the project started, children living in a geographical area belonged to a specific public school district with preschool and elementary classes. The children started in their preschool class and continued in the same school with the same school mates until grade 9. In senior high they went on to programs according to their interests and future plans. In Sävsjö when the project started there were six different school districts (six different municipal schools with eight preschool classes and grades 1–3). Three of the schools were located in the main town and the others in three smaller suburbs.

Since the children in each geographical area belonged to their own school district, they were placed by the school authority in the school and the class to which they belonged. Due to these circumstances the clustering was given through the students’ residential area and school affiliation. By lot, one school in one of the suburbs and two schools in the main town (with four individual preschool classes) become “experimental schools” to test the alternative curriculum, while the other three (also with four individual preschool classes) were the “comparison schools”. Both in the experimental group and in the comparison group there were 4 individual classes with approximately 24 pupils in each class. In the main town, mixed-age classes had been introduced earlier. In a leaflet to parents, the local school authority claimed that mixed-age classes had organizational benefits, such as: only half of the children are new each year, classes are uniform; there is more similarity and more community within the work unit; there are several group lessons and an increased adult density. Furthermore, with children teaching each other, they take greater responsibility for their own learning, thus each student can work at his/her own pace and according to his/her own situation. By lot, almost half of the children in the experimental classes came to mixed-classes when starting grade 1. They were to spend their first 6 years in school in mixed-age classes.

The free school choice introduced in Sweden in the 1990s means that parents and students can themselves choose the school the pupil should attend. The free school choice had not been put into use in Sävsjö when the study was conducted. Had this been the case, the investigation could not have been carried out in this way because the current selection procedure could not have been used and most probably the students may have changed school and class over the period of the study.

#### Subjects

184 families with 186 6-year old children (including 2 pairs of twins, two girls in one of the intervention classes and 2 boys in one of the comparison classes) started preschool in August 1998. They were invited to take part in the study. One family moved just before school-start and 8 decided not to participate, which is why only 177 children entered the study. Their mean age was 6.2 years (range 5.6–6.6 years). 92 children (44 boys and 48 girls) entered the experimental classes and 85 children (40 boys and 45 girls) entered the comparison classes. A comparison between the groups was made by addressing the children’s birth months over the year they were born. No statistically significant difference was found.

Over the four school years, 7 children in the experimental group (including the pair of twins), and 13 children in the comparison group, moved away from the city. In total, 19.3% of the children moved away from the community. 85 children (40 boys and 45 girls) in the experimental group and 73 children (31 boys and 42 girls) in the comparison group still lived in the city at end of grade 3 (spring 2002) and had taken part in the project.

Data on the fathers´ education and occupation (given voluntarily in 1999 and 2002) showed that seven (all belonging to the experimental groups but living in three different geographical areas with their children in three different classes), had a university degree, seven were entrepreneurs, twenty-four had vocational training with at least 3 years of after upper secondary school study, one hundred and five had completed upper secondary school and the remaining thirty four had minor training according to the “skill levels” provided by SSYK-96 [23]. SSYK-96 (Standard för svensk yrkesklassificering (Statistiska centralbyrån, 2001) was based on ISCO-88 [24], The International Standard Classification of Occupations. No statistically significant difference was found.

### Principles of the educational intervention

Within the frame of this project, an alternative curriculum was set up that integrated the preschool year with the first three school years of elementary school. The basic idea was to use “differentiation within the class” to allow individual teaching and give each pupil the same chances for social participation, i.e. to be integrated/included in the class’ “social community” and to share the same level of average knowledge, i.e. to be part of “knowledge community” of the class to avoid exclusion.

One of the preschool teachers followed the class to elementary school as a coordinator in order to facilitate the children´s social function in the class, handle individual teaching and, if necessary, give opportunities to divide the class into teaching groups. The focus of the educational activities in the preschool year was regular school preparatory elements and providing social support to function in groups. The children’s linguistic awareness was a key interest for the intervention. At the start of the preschool year, a screening with a language test was performed. The results showed a great variation of language competence among the children and every 4th child failed to reach the expected average language competence for “children starting the preschool year”. For the experimental group, the findings from the language screening were used for individual training of language and concepts.

A logbook was set up (see Appendix) to ensure that each child each day in a playful way took part in the educational activities and had social support if needed. The project, the preschool activities and the curriculum have been well described in Swedish [1, 2].

The principles behind the educational intervention used can be summarized as follows:

- There was a high level of educational stimulation in the pre-school class, in order to prepare all students for the first school year. A work plan was drawn up with the topics “Ethics and Morals”, “Mathematics”, “Music”, “Nature Orientation “, “Moving and Sport”, “Social Science “, “Creative Subjects” and “Swedish”;
- A log book was set up for preschool year I to be used to ensure that each pupil, individual or in groups, was every day stimulated according to the work plan. See Appendix.
- There was a great emphasis on linguistic awareness, reading and writing. A language test was performed at the start of the preschool class to screen for language skills and deficits. The topic “Swedish” in the work plan was divided into the following steps: language games, books, speech, writing, and drama and computer management. “Language games” meant dealing with rhymes, chants, sentences, syllables, synthesis/segmentation, compound words, classification, articulation, comparison of word length and absurdities/riddles.
- There was a great emphasis on socio-emotional aspects in preschool in order to lay the foundations for a positive attitude towards learning and collaboration with other students and teachers. This part of the work plan was subtitled “Ethics and Morals”, which was designed to teach students to show respect and understanding for others, be honest and tell the truth, treat others as you want to be treated, emphasize the equal value of individuals, understand what is right and wrong and distinguish between mine and yours.
- The planning of learning activities was made with awareness of each student’s maturity level in order to avoid experiences of stress and/or failure by the student. For a long time, the concept of “global” maturity has been used in Sweden in relation to child development to indicate the difference between mental age and chronological age as measured by IQ-tests with respect to children’s normal growth and their school achievements. Swedish parents are familiar with this concept of “maturity”. To measure maturity the parents assessed their children on two items [25] as follows: In the first question, parents compared their child’s level of maturity to an average child of the same age on a 5-point likert scale (1 = very mature, 2 = somewhat mature, 3 = average, 4 = somewhat immature and 5 = very immature). In the second item, parents estimated their child’s perceived age, independently of chronological age. From grade 1, the learning process was to be monitored by observation and periodic testing. The results were to be used for groupings in classes and those who were found weakest at the last measurement were to receive the most attention.
- From school start, the functional assessments of the students’ development in the areas of behavior and cognition were to be followed by IQ-tests, the monitoring of reading and mathematical skills using standardized tests, and questionnaires to parents, the teacher and students.
- The overlapping of teachers in the pre-school class and in school was planned: one pre-school class teacher should follow the students during their three school years, and the schoolteachers (who received the pupils in grade 1) should do part of the pre-school class teaching. This meant a slightly increased staffing ratio.
- Increased didactic continuity between pre-school class and school class was planned.
- It was decided that enhanced teacher resources should exist, as the experimental classes together had three coordinators sharing two full-time positions.
- The teaching of mathematic content was planned to be postponed until the students had reached a certain linguistic and conceptual maturity.
- It was determined that teachers should have access to child psychiatric counseling, which meant confirmations of teachers’ observations and assessments, but also a greater understanding and changing perspectives on student behavior.

### Training of teachers and classroom work

All teachers in the city’s preschools and grades 1–3 classes, irrespective of responsibility for experimental or comparison groups, had the same basic training about the project and the aims. Joint lectures were given in relevant fields of knowledge such as neuropsychology, the relationship between education and health, the concepts of reading and writing difficulties and problems with learning mathematics. Olof Magne [12] and Ewe Malmquist [13], two senior researchers and experts in dyscalculia and dyslexia were part of the faculty lecturing for the teachers. The teachers in the experimental group were to have continuous support over the 4 years from a child and adolescent psychiatrist to discuss how to solve problems referring to pupils’ behavior, home situation and learning problems. The child and adolescent psychiatrist worked according to the “old knowledge” from Swedish school psychiatry, which in many aspects is a much broader concept compared to today’s current understanding of “child neuropsychiatry”.

The project in the experimental classes was to be guided and led parallel to the regular organization. The individual school had to concretize the activities as an extra mission. Regular staff was to carry out the project, and extra resources were provided in the form of preschool teacher “coordinators” who followed their pupils during the first years of elementary school. The coordinators were to be responsible for the project at the school. By coordinators following the students throughout the project, the knowledge of each student and the project’s progress could be recorded. When not working on their administrative duties, coordinators were to assist in the classes both with teaching and social activities. In addition to basic staffing, which included one teacher per class and the coordinator, pre-school teachers, special education teachers, other teachers, as well as assistants and leisure-time teachers were to be involved. The in-depth knowledge of the students that was gathered covered both the experimental classes and the comparison classes but was only to be used for the planning of teaching and social activities in the experimental classes. The staffs in these classes were to have access to child psychiatric advice throughout the project. If needed, the experimental classes could be divided into smaller groups and individual teaching could be given.

### The teaching principles of the project

The project was to use a modern concept of the Swedish teaching principles from the period 1946–1970. Based on previous knowledge of Swedish education and school mental health, and knowledge of the considerable variation in learning age with relation to chronological age, these principles can be summarized as follows:

#### To support

Pupils’ creativity, language and speech competence and social competence.

#### To accept

Each pupil’s individual level of maturation/developmental level and behavior.

#### To introduce alternative curricula for

Talented children, children with school-immaturity, slow learning capacity, mental retardation, etc.

#### To introduce special training for teachers

In order to know how to teach children with “problems”.

#### To use screening and monitoring of skills

Screen for intellectual skill, language, reading, spelling, maths, maturity, behavior, health at preschool start, the “School-maturity test” and monitor achievements over the school years.

### The measures

At preschool start, parents filled in a questionnaire concerning their opinions of their children’s maturation [25], behavior and reaction when he/she was sad, afraid, anxious, irritated, etc. Based on this information, the teachers in the experimental group should be able to adapt individually to each child’s level of maturation and not misinterpret the children’s behavior, especially boys who react with restlessness and aggressive behavior when sad or disappointed.

At school-start, all children were tested using the same tests. An IQ-test (the SPIQ-test) [26] was performed and administered in the class-room, making it possible to test all children at the same session. The Umesol-test—“Listen to the words” [27] was used to assess language competence and phonological awareness.

When the project ran, no modern standardized Swedish test existed to measure skills in mathematics in the lower grades. However, Olof Magne [12] (one of the Swedish professors in education with a special interest in special educational needs in mathematics) was a consultant for the project. His book “Den nya specialpedagogiken i matematik—En utmaning i läroplanstänkande” [28] was used in the planning of teaching mathematics. One of the teachers in the project was undergoing training in special education at Malmö Högskola with Magne as supervisor. She developed a test for the study [29], based on the included Sävsjö children, as part of her examination as a special pedagogue. This test was used to compare mathematical skills between the groups.

During the three first years in elementary school, the children’s reading capacity was monitored using Läskedjor [30], Lindahl’s Högläsningsprov H4 [31]. At end of grade 3, DLS [32, 33] was used.

At end of grade three, the children themselves made self-reports of their school satisfaction, i.e. their attitudes towards learning and collaboration with other pupils. A self-report questionnaire with 21 items on skills/desire for school work and social adjustment/competence using a Likert scale in the form of pictograms with 5-steps was developed for the project and used for the self-evaluation at end of grade 3. In this way, the children reported their confidence in reading, writing, etc., using the Likert scale’s five steps. For each item “goes very well” was the fifth step, the maximum.

The Child Behavior Checklist (CBCL) by Thomas Achenbach [34, 35] was used. Parents (parent rating scales) and teachers (teacher rating scales) filled in the questionnaires at school-start and at end of grade 3. When comparing parents’ and teachers’ questionnaires at the end of grade 3, it was found that parents’ and teachers’ assessments were quite comparable except in two of the comparison groups. In these two comparison groups, the parents assessed their children as having more internalizing and externalizing symptoms compared to the teachers’ assessments.

At school-start and at the end of grade 3, parents and teachers filled in a questionnaire [36] on AD/HD using the criteria of DSM-IV in yes/no-alternatives.

**Fig. 1:**
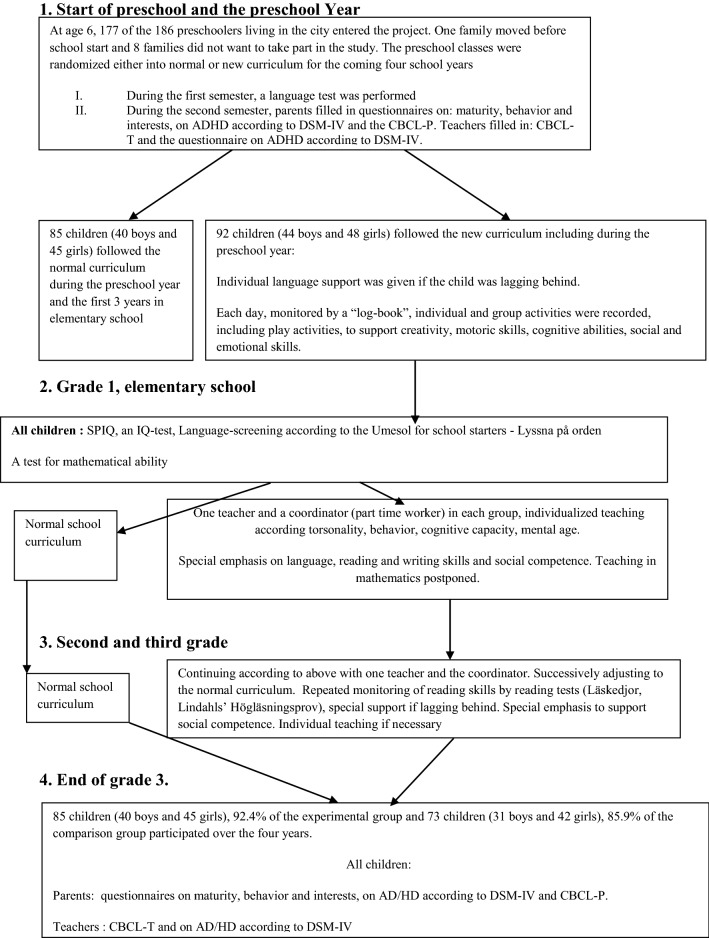
The School-project—overview of design, data collection, educational and preventive measures until end of grade 3

Figure 1 the design of the intervention.

### Statistical analysis

The data analysis has been conducted using the statistical software program Stata. All data was computerized and the programs Stata 15 software [37] and SAS/STAT^®^ software (Version 9.4, SAS Institute Inc) [38] were used for calculations. Descriptive statistics were used. However, in order to test if the interventions did per se improve the children’s reading achievements and overall satisfaction, regression analyses were performed. Details of the statistical analyses used are given at the end of each table.

Multilevel analysis was not used here due to a small sample. In addition to data on IQ-values, no true pre-intervention information was available to measure the effect of the intervention using pre- and post intervention data. There were a few single children who could read at school-start, but there were no differences between the groups when it came to language competence and phonological awareness at school-start. Because of this, the great variation of normal development in children starting school and the intervention’s aim to support and monitor language awareness and reading and writing skills, it seems reasonable to measure the outcome as done by comparing the groups at the start and at the end of consecutive school years.

### Ethical vetting and permissions

Ethical permission for the project, and its continuation until the pupils left senior high, was given by the Ethical Committees of Linköping’s University and the Karolinska Institutet, Dnr: 99-141 and by the Regional Ethical Review Board in Stockholm, 2013/1062-32.

## Results

### Language competence and phonological awareness at school-start (age 7 years)

The overall correlation quotient between SPIQ and UMESOL was 0.16. There were no statistically significant differences regarding UMESOL or SPIQ between the intervention group and comparison group when controlling for age and gender (see Table 1). However, at school start there was a difference in IQ, experimental group < comparison group, p = 0.006, while no difference was found at end of grade 3.

**Table 1:** Baseline measures: Umesol and SPIQ at the beginning of the school start

	SPIQ				Umesol			
	Score		Stanine		Score		Stanine	
	M (SD)	Min–max (n)	M (SD)	Min–max (n)	M (SD)	Min–max (n)	M (SD)	Min–max (n)
Experimental group								
Boys	19.5 (2.7)	12–25 (43)	3.6 (1.6)	1–6 (43)	20.6 (4.9)	10–25 (44)	6.0 (2.0)	2–8 (44)
Girls	19.8 (2.6)	13–25 (48)	3.8 (1.7)	1–6 (48)	23.1 (3.4)	4–25 (47)	6.7 (1.4)	1–8 (47)
Total	19.6 (2.7)	12–25 (91)	3.7(1.6)	1–6 (91)	21.9 (4.4)	4–25 (91)	6.4 (1.7)	1–8 (91)
Comparison group								
Boys	21.2 (2.8)	13–28 (33)	4.5 (1.4)	1–6 (33)	21.3 (4.7)	4–25 (32)	5.7 (1.8)	1–8 (32)
Girls	20.7 (3.7)	8–27 (44)	4.3 (1.6)	1–6 (44)	21.3 (4.5)	9–25 (44)	6.0 (1.7)	2–8 (44)
Total	20.9 (3.3)	8–28 (77)	4.4 (1.6)	1–6 (77)	21.0 (4.6)	4–25 (76)	5.9 (1.7)	1–8 (76)

Differences were evaluated with a linear regression, total score was the dependent variable and covariables were age and gender. The only significant difference was IQ (experimental group < comparison group, p = 0.006)

### Capacity to read at the end of grade 1 and at end of grade 3

The capacity to read was monitored over the first three grades. Differences were found at the start of grade 2, using “Läskedjor”, where the intervention group performed better on word chains while the comparison group performed better in spelling. These differences had disappeared at start of grade 3.

At end of grade 1, boys and girls in the experimental groups had better reading achievements according to Lindahl’s högläsningsprov, a difference that increased every year until end of grade 3. Separate regression analyses were performed for each test, where the test result was modeled as a function of experimental/comparison schools (*p* value), sex, age and IQ (the mean of the two individual SPIQ-values from each pupil). Repeated measurement with ANOVA using all 5 individual measurements according to the Lindahl’s högläsningsprov showed significant differences, as the intervention group performed better than the comparison group (p = 0.002, es = 4.35). See Table 2.

At end of grade 3, the experimental classes had better reading comprehension according to the DLS-test (p = 0.03, es = 0.04). No differences were found with reference to mathematical skills. See Table 2.

**Table 2:** Reading capacity at the end of grade 1, grade 2 and grade 3. Mathematical capacity at end of grade 3

Test used	Age	Group						
		Experimental			Comparison			p-value
	M (SD)	Mean	SD	n	Mean	SD	n	
Läskedjor (school start grade 2)								
Word chains (stanine)	8.3	5.2	2.1	84	4.4	1.9	74	0.004
Letter chains (stanine)		4.5	1.7	84	5.0	1.8	74	ns
Spelling (stanine)		4.5	1.6	84	5.1	2.0	74	0.04
Läskedjor (school start grade 3)								
Word chains (stanine)	9.3	3.7	1.6	26	4.5	1.9	29	ns
Letter chains (stanine)		4.8	1.8	26	5.3	1.9	24	ns
Spelling (stanine)		5.0	1.7	83	4.8	1.7	73	ns
Lindahls högläsningsprov (correct no of words/min)								
End of grade 1	7.9	46.4	19.2	89	41.5	18.4	78	0.02
School start grade 2	8.3	55.8	20.8	64	49.1	18.6	67	0.04
End of grade 2	8.9	76.8	17.6	62	65.1	19.9	60	0.000
School start grade 3	9.3	83.6	19.9	59	71.5	20.7	60	0.001
End of grade 3	9.9	91.4	18.7	84	82.5	19.5	74	0.002
DLS-test (end grade 3)								
Correct sound	9.9	37.9	2.7	84	37.7	2.1	74	ns
Correct spelling		16.6	2.7	84	16.7	3.2	74	ns
Vocabulary		13.3	3.2	84	13.5	2.8	74	ns
Reading comprehension		14.5	3.0	84	14.4	2.2	74	0.03
Maths (end grade 3)	9.9	72.8	12.5	83	71.8	12.5	74	ns

Sweden has a two semester school year. It starts with the autumn term (Ht) and ends with the spring term. Differences were evaluated with a linear regression; test score was the dependent variable and covariables were age, gender and IQ (the mean of the two individual SPIQ-values from each pupil). ANOVA using all 5 individual measurements according to the Lindahl’s högläsningsprov showed significant differences, experimental classes > comparison-classes (p = 0.002)

### Self-evaluation of skills and adjustment at the end of grade 3

A self-report questionnaire, utilizing a Likert scale with 21 questions on skills/desire for school work and social adjustment/competence, was used. The children evaluated their own skills i.e. reported their confidence in reading, writing, etc., using the Likert scale’s five steps. For each item, “goes very well” was the fifth step, the maximum. Ordered logit estimation was performed for each question to control for the children’s sex. The p-values reported refer to the comparisons between the experimental and comparison schools when the whole Likert-scale was used in the analyses to control for sex. Significant differences were found for the following items (experimental group > comparison group): “To read goes very well” (p = 0.02, es = 0.23); “To write goes very well” (p = 0.007, es = 0.35); “To do mathematics goes very well” (p = 0.003, es = 0.48). The item “Seldom/never being teased” showed a different outcome as the comparison group rated themselves in favor of the experimental group (See Table 3).

**Table 3:** Self-evaluation of skills and adjustment at end of grade 3

Item answered	Experimental group (%)	Comparison group (%)	p-value
Reading goes very well	57.1	38.9	0.02
To write goes very well	50.0	26.4	0.007
To do mathematics goes very well	56.0	34.7	0.003
Seldom/never being teased	66.7	80.6	0.02

Differences were evaluated with an ordered-logit estimation. Answers in a Likert-scale were the dependent variables and p-values reflect adjustment for sex

### Behavioral problems according to the Child Behavior Checklist (CBCL)

Both parents and teachers filled in the CBCL at school-start and at end of grade 3. The children in the comparison group showed more symptoms of anxiety and depression (p = 0.031) at school-start. At the end of grade 3, no differences were found (Table 4).

**Table 4:** Levels of CBCL-syndromes (parental assessments) at 7 and 10 years of age in experimental and comparison schools

CBCL	7 years of age			10 years of age		
	Experimental group (n = 70)	Comparison group (n = 61)	p*	Experimental group (n = 72)	Comparison group (n = 58)	p*
	Mean (SD)	Mean (SD)		Mean (SD)	Mean (SD)	
Syndromes of problems						
Withdrawn	1.06 (1.09)	1.49 (1.15)	ns	1.32 (1.77)	1.02 (1.24)	ns
Somatic complaints	0.66 (1.15)	0.74 (1.05)	ns	0.96 (1.49)	0.81 (1.07)	ns
Anxious and depressed	1.49 (1.77)	2.71 (1.63)	0.031	1.97 (2.43)	1.88 (2.46)	ns
Social problems	0.74 (1.13)	0.82 (1.49)	ns	0.82 (1.47)	0.64 (1.39)	ns
Thought problems	0.07 (0.31)	0.07 (0.25)	ns	0.11 (0.46)	0.07 (0.26)	ns
Attention problems	1.16 (1.47)	1.57 (2.03)	ns	1.44 (1.93)	1.28 (2.09)	ns
Delinquent behaviour	1.00 (1.33)	1.07 (1.33)	ns	0.92 (1.34)	0.57 (0.82)	ns
Aggressive behaviour	5.07 (4.61)	4.95 (4.12)	ns	4.63 (4.74)	3.62 (3.86)	ns
Grouping of syndromes						
Internalising	3.20 (3.27)	4.52 (4.48)	0.049	4.21 (4.56)	3.67 (3.70)	ns
Externalising	6.07 (5.56)	6.01 (5.02)	ns	5.54 (5.67)	4.19 (4.33)	ns
Total behaviour problem score	16.1 (10.87)	17.90 (12.04)	ns	15.89 (12.33)	13.59 (10.36)	ns

p*: p-values correspond to t-tests

### The intervention effect on reading capacity from grade 1 to grade 3

For 89 children in the experimental group (42 boys and 47 girls), and 74 children in the comparison group (31 boys and 43 girls), complete data existed for the Lindahl’s Högläsningsprov and for the whole test period from grade 1 to grade 3, making it possible to test if the interventions had an effect on the children’s reading capacity. At the end of grade 1 (Spring 1999), there were no statistically significant differences regarding reading capacity between the two groups when controlling for age and sex, while at the end of grade 3 statistically significant differences (p < 0.01) were found in favor of the children in the intervention group (Table 5).

**Table 5:** Reading capacity at the end of grade 1 and grade 3. A regression analysis using the results from the Lindahl’s Högläsningsprov

	Score grade 1		Score grade 3	
	M (SD)	Min–max (n)	M (SD)	Min–max (n)
Experimental group				
Boys	43.6 (20.7)	6–102 (42)	88.6 (19.9)	47–121 (39)
Girls	48.9 (17.7)	17–86 (47)	93.9 (17.3)	50–127 (45)
Total	46.4 (19.2)	6–102 (89)	91.4 (18.7)	47–127 (84)
Comparison group				
Boys	40.9 (18.9)	12–100 (33)	79.4 (21.0)	46–153 (31)
Girls	41.9 (18.2)	10–108 (45)	84.8 (18.2)	50–135 (43)
Total	41.5 (18.4)	10–108 (78)	82.5 (19.5)	46–153 (74)

At the end of grade 1 there were no statistically significant differences regarding reading capacity between the index group and comparison group when controlling for age and gender (p-value = n.s.). At the end of grade 3 there were statistically significant differences (p < 0.01) regarding the reading capacity between the experimental and comparison group when controlling for age and gender. Linear regression was used in Stata with the total score as a dependent variable and the independent variables group (Experimental/Comparison), age and gender

## Discussion

The findings support the assumption that the curriculum used, based on “old principles” and individualized teaching, gave the pupils better reading and writing skills when leaving the first 3 years of elementary school. They liked mathematics and school activities better than those in the comparison group, although they experienced teasing more often than the comparison group. IQ-differences that existed at school start had disappeared at end of grade 3. No difference existed in relation to CBCL at end of grade 3. One child in the comparison group fulfilled criteria for AD/HD according to parents and teachers. The results of the self-evaluation indicate that the children in the experimental group felt part of the class’ “social community” and “knowledge community”.

How should the main results be explained? Based on previous knowledge of when Sweden had a differentiated school and active integrated school psychiatry, these findings are not surprising. That IQ can change over time (in average an incline with 15 IQ-points can be expected) has been well documented both previously [16, 17, 19, 20] and in modern times [21]. That an individualized teaching based on the child’s cognitive capacity is good for the child is also well documented. It is most probable that the ideas behind this school experiment “differentiation within the class”, based on the concept of an inclusive school that identified children with cognitive problems and supported them individually, were good. It is also probable that the information the parents gave about their children’s global maturity and “strengths and difficulties” was important for the teachers in their daily work and helped them to not misinterpret pupils’ behavior when the children were stressed.

In the differentiating Swedish school, a special curriculum was offered to children with slow learning abilities (IQ = 70–90), attention problems and weak working memory, i.e. symptoms and problems that are today called AD/HD. In the experimental classes, the pupils’ problems with inattention and restless behavior became very mild when targeted with educational strategies, which may explain why the symptoms very seldom became a functional impairment for them.

The strategy within the project was to deal with executive weaknesses as “was done before” [6, 14]. This is very much in line with Rosemary Tannock’s proposal “Reconceptualizing AD/HD” in 2001 [39]. She proposed that “New findings suggest that AD/HD is a learning disorder rather than a behavior disorder. Thus teaching strategies that target cognitive weaknesses may be more effective than behavioral management techniques in promoting academic success for students with AD/HD”. This is also in line with the knowledge from previous Swedish school-psychiatry [9, 10]. Although AD/HD existed in those days under the names of Cerebral Damage and later MBD (minimal brain damage/dysfunction), the children’s problems seldom became so serious that teachers and parents could not manage them. In the current project, both parents and teachers assessed AD/HD-symptoms according to DSM-IV [36]. In the experimental classes, no child fulfilled criteria for AD/HD at end of grade 3 while only one child in the comparison classes did, which supports Tannock’s recent proposal [39].

The tests used are all standardized Swedish tests. As they are relatively unknown outside of Sweden, they are commented on as follows: To monitor and test reading capacity and achievements, the Umesol-test [27]—a modern test measuring phonological awareness—and three other tests, “Läskedjor” [30], the “Lindahl’s Högläsningsprov” [31] and the DLS-test [32, 33] were used in the project. All three tests are standardized Swedish tests. Lindahl’s test and the DLS (with the first version presented in 1945) were used in Swedish schools from 1940s to 1970s, when there were special programs in Swedish schools to support pupils with reading and writing difficulties and dyslexia. The interest to use them in Swedish schools then disappeared for almost 20 years, but from the mid-1990s they were introduced again.

In 2014, SBU, the Swedish Agency for Health Technology Assessment and Assessment of Social Services presented the report: “Dyslexia in Children and Adolescents-Tests and Interventions” [40]. Acceptable reliability was found for the Umesol-test. Acceptable reliability and validity were found for Läskedjor and the modern version of DLS, while the Lindahl’s Högläsningsprov was not fully assessed, probably due to its “old age”. For this project it was used because it is well-known and provided an opportunity to compare reading skills among school-starters up until the 1970s when it was widely used in Sweden.

The SPIQ- test [26] is a Swedish standardized IQ-test for schoolchildren administered for group-testing. It was chosen for the project as it could be administered in the classroom to test all children during the same session.

The CBCL-questionnaire [34, 35] (based on the parental assessments) was used to assess behavioral problems among the children. The difference found that the children in the comparison group showed more anxiety/depression at school start compared to the experimental group. This could perhaps be explained by the efforts in the experimental preschool classes to prepare the children for school or perhaps also by the fact that the teachers in the preschool classes had support from a child and adolescent psychiatrist to discuss individual problems among pupils.

From the diaries filled in by the coordinators during the project period, there is information indicating that the teachers in the experimental groups felt their daily work as teachers was both meaningful and inspiring. This is an interesting finding and needs to be further explored. There is also information that suggests that parents whose children had been diagnosed with mental retardation or so-called borderline IQ found that their children were unable to keep up with the schoolwork in upper grades and decided to move their children to classes for special education.

### Strengths and limitations

The present study, a cluster-randomized trial, has both strengths and limitations. The strength is that the project included 95% of the city’s 6-year old children at start, divided into an experimental group and a comparison group with few drop-outs (one family moved just before school-start and 8 decided not to participate), following a strict design with an educational intervention. The project management could not influence the randomization because the division of the children into the respective classes was due to the municipality’s organization and their living area.

The limitations are the overall small sample with 19.3% of the children moving out of the community during the intervention. However, this group will be followed up at end of grade 12 as will all other children.

There is also a possible teacher-bias. The teachers in the comparison classes had the same basic information about the ideas behind the project and took part in the same seminars preparing for the project as the teachers in the experimental classes, which could affect the teachers in the comparison classes and their ambition that their children should also learn to read well.

When the project started, the intention was to run a “sister-project” in one of the bigger Stockholm suburbs where the population in 1998 was around 7 times greater than that of Sävsjö. This should have provided an adequate sample of 6-year old children to include in the project. However, the change in the Swedish school-system that introduced “the free school choice” made it impossible to keep the suburb’s cohort of 6-year old children together over 4 years and randomize them according to an intervention design.

### The findings in relation to current opinions

In 2010, the Health Committee at The Royal Swedish Academy of Sciences [41] initiated a State of the Science Conference to address the following issues: “School, Learning and Mental Health”. The ideas behind the Sävsjö-project are well in line with a conclusion provided by the conference: “Early school failures and in particular reading difficulties cause internalizing and externalizing mental health problems”.

The results are of interest in relation to the recently presented Governmental Public Investigations from the Swedish School Commission in 2016 [42, 43] and 2017 [44]. Each of them, along with English summaries, is available on the governmental website: http://www.government.se/.

OECD [45] has commented on the School Commission work in a recent report called Improving Schools in Sweden: “Sweden’s disappointing performance on PISA has sparked the national debate on the quality and future of education in Sweden which seems to have resulted in a broad consensus on the need for change and support for the various school reforms and policies that the Swedish government has embarked on in recent years.”

Since some of the ideas presented by the Commission were included in the Sävsjö-project, it seems relevant to go on with these ideas from the Commission to provide Swedish pupils better teaching, schooling and better results in the PISA-investigations.

It is difficult to relate the study and the results to countries outside Sweden. The long history of “School Psychiatry as a branch of Child and Adolescent Psychiatry” is rather unique for Sweden, as School Psychiatry focused on the relationships between learning, education and mental health. A similar approach can be found in France in the work of Alfred Binet, as well as in Switzerland where “Heilpädagogie” was a university discipline in Zürich. There are also modern textbooks on School Mental Health. One example is “Handbook of School-based Mental Health Promotion—An Evidence-Informed Framework for Implementation” from 2018 [46], although it covers other aspects of mental health in addition to its relation to education and learning. Besides Rosemary Tannock’s paper [39] from 2001 “Reconceptualizing AD/HD” when she proposed that “New findings suggest that AD/HD is a learning disorder rather than a behavior disorder” you will find few papers on such topics. One is Whitsell’s paper [47] from 1969 “Learning disorders as a school health problem. Neurological and psychiatric aspects” where he discusses the need for “improved specific remedial educational techniques”. Another paper is by Noam and Hermann from 2002 [48] discussing “Where education and mental health meet”. They present “a school-based prevention and intervention method for young adolescents called Responsive Advocacy for Life and Learning in Youth (RALLY). Prevention practitioners, a new role developed by the program, work in classrooms and after-school settings to provide non stigmatizing support to students. Using a three-tiered prevention model, practitioners integrate a mental health and educational focus to foster students’ academic, social, and emotional success.”

There are similarities between “Rally” and our project, as one of the ideas behind it is to give children a fair chance to feel and be part of the class’ “social community” and “knowledge community” to support their self-confidence and self-image and avoid exclusion and school failure, which in our opinion will minimize the development of school mental health problems [49]. The self-evaluation of academic achievements at the end of grade 3 supports such a possibility, as the intervention group felt more often that reading, writing and mathematics went well.

### Further research

This part of the project should be considered as the starting point and basis for reporting the results of the follow-up that was performed when the students completed senior high school. The aim of the follow-up will be to investigate whether the efforts put into the children’s education during their preschool year and at school-start have given them a better ground for later achievements that result in observable educational attendance and success. Other aims for further analyses will be to follow the outcomes of those children in the experimental classes who were slow learners and to compare the results of children who attended “age mixed-classes” with those of “same year- classes”.

## Conclusion

The alternative curriculum covering the preschool year through the first 3 years of elementary school and based on old principles from the curative education (“Heilpädagogie”), educational psychology and school psychiatry used in the Swedish public school system until the 1970s gave today’s children in the experimental classes a better reading capacity and better reading comprehension compared to the children in the comparison group who followed the average school curriculum. However, as the sample is small, and as the city of Sävsjö is a small city, the findings cannot be generalized. They should, however, be seen as a promising opportunity to develop education in Swedish schools.

## Figures and Tables

**Fig. 2:**
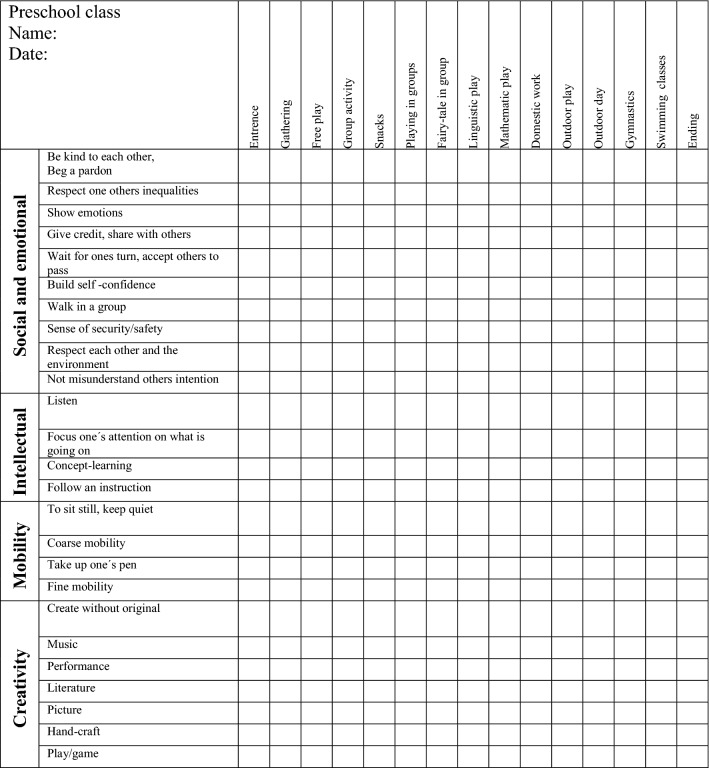
Log-book used daily during the year in pre-school classes. Every day interventions were evaluated and the boxes indicated either by check or by time to indicate a specific task completed.Guided by this ensuing operations could be planned so that they best benefited the children's group, the individual child and the goals

## Data Availability

All data and the material are available at the Sävsjö City Council and at the Karolinska Institutet in Stockholm.

## Appendix

See Fig. 2.

## Abbreviations

ANOVA: analysis of variance
CBCL: Child Behavior Checklist
DSM-IV: Diagnostic and Statistical Manual of Mental Disorders
SPIQ: A Swedish IQ-test developed for group-testing in school-classes
Umesol: “Listen to the words”, a Swedish test to measure phonological awareness, reading and spelling capacity and self-image among children in the first grades of elementary school
H4: A Swedish test, “Lindahl’s Högläsningsprov” to test for word decoding and read speed among school children
DLS: A Swedish test developed to assess reading comprehension, vocabulary, spelling and phonological awareness
SOU: The Swedish government’s official investigations
ISCO: The International Standard Classification of Occupations
SSYK 96: Standard för svensk yrkesklassificering
OECD: Organisation for Economic Co-operation and Development,
PISA: Programme for International Student Assessment
